# Fundamentals and Recent Developments in Approximate Bayesian Computation

**DOI:** 10.1093/sysbio/syw077

**Published:** 2016-09-11

**Authors:** Jarno Lintusaari, Michael U. Gutmann, Ritabrata Dutta, Samuel Kaski, Jukka Corander

**Affiliations:** 1 *Department of Computer Science, Aalto University, Espoo, 00076, Finland; Helsinki Institute for Information Technology HIIT*; 2 *Department of Mathematics and Statistics, University of Helsinki, Helsinki 00014, Finland; Helsinki Institute for Information Technology HIIT*; 3 *Department of Biostatistics, University of Oslo, 0317 Oslo, Norway*

## Abstract

Bayesian inference plays an important role in phylogenetics, evolutionary biology, and in many other branches of science. It provides a principled framework for dealing with uncertainty and quantifying how it changes in the light of new evidence. For many complex models and inference problems, however, only approximate quantitative answers are obtainable. Approximate Bayesian computation (ABC) refers to a family of algorithms for approximate inference that makes a minimal set of assumptions by only requiring that sampling from a model is possible. We explain here the fundamentals of ABC, review the classical algorithms, and highlight recent developments. [ABC; approximate Bayesian computation; Bayesian inference; likelihood-free inference; phylogenetics; simulator-based models; stochastic simulation models; tree-based models.]

## Introduction

Many recent models in biology describe nature to a high degree of accuracy but are not amenable to analytical treatment. The models can, however, be simulated on computers and we can thereby replicate many complex phenomena such as the evolution of genomes (Marttinen et al. 2015), the dynamics of gene regulation (Toni et al. 2009), or the demographic spread of a species (Currat and Excoffier 2004; Fagundes et al. 2007; Itan et al. 2009; Excoffier et al. 2013). Such simulator-based models are often stochastic and have multiple parameters. While it is usually relatively easy to generate data from the models for any configuration of the parameters, the real interest is often focused on the inverse problem: the identification of parameter configurations that would plausibly lead to data that are sufficiently similar to the observed data. Solving such a nonlinear inverse problem is generally a very difficult task.

Bayesian inference provides a principled framework for solving the aforementioned inverse problem. A prior probability distribution on the model parameters is used to describe the initial beliefs about what values of the parameters could be plausible. The prior beliefs are updated in light of the observed data by means of the likelihood function. Computing the likelihood function, however, is mostly impossible for simulator-based models due to the unobservable (latent) random quantities that are present in the model. In some cases, Monte Carlo methods offer a way to handle the latent variables such that an approximate likelihood is obtained, but these methods have their limitations, and for large and complex models, they are “too inefficient by far” (Green et al. 2015, p. 848). To deal with models where likelihood calculations fail, other techniques have been developed that are collectively referred to as likelihood-free inference or approximate Bayesian computation (ABC).

In a nutshell, ABC algorithms sample from the posterior distribution of the parameters by finding values that yield simulated data sufficiently resembling the observed data. ABC is widely used in systematics. For instance, Hickerson et al. (2006) used ABC to test for simultaneous divergence between members of species pairs. Fan and Kubatko (2011) estimated the topology and speciation times of a species tree under the coalescent model using ABC. Their method does not require sequence data, but only gene tree topology information, and was found to perform favorably in terms of both accuracy and computation time. Slater et al. (2012) used ABC to simultaneously infer rates of diversification and trait evolution from incompletely sampled phylogenies and trait data. They found their ABC approach to be comparable to likelihood-based methods that use complete data sets. In addition, it can handle extremely sparsely sampled phylogenies and trees containing very large numbers of species. Ratmann et al. (2012) used ABC to fit two different mechanistic phylodynamic models for interpandemic influenza A(H3N2) using both surveillance data and sequence data simultaneously. The simultaneous consideration of these two types of data allowed them to drastically constrain the parameter space and expose model deficiencies using the ABC framework. Very recently, Baudet et al. (2015) used ABC to reconstruct the coevolutionary history of host–parasite systems. The ABC-based method was shown to handle large trees beyond the scope of other existing methods.

While widely applicable, ABC comes with its own set of difficulties, that are of both computational and statistical nature. The two main intrinsic difficulties are how to efficiently find plausible parameter values and how to define what is similar to the observed data and what is not. All ABC algorithms have to deal with these two issues in some manner, and the different algorithms discussed here essentially differ in how they tackle the two problems.

The remainder of this article is structured as follows. We next discuss important properties of simulator-based models and point out difficulties when performing statistical inference with them. The discussion leads to the basic rejection ABC algorithm which is presented in the subsequent section. This is followed by a presentation of popular ABC algorithms that have been developed to increase the computational efficiency. We then consider several recent advances that aim to improve ABC both computationally and statistically. The final section provides conclusions and a discussion about likelihood-free inference methods related to ABC.

## Simulator-based Models

### Definition

Simulator-based models are functions $M$ that map the model parameters $\theta$ and some random variables $V$ to data $y$. The functions $M$ are generally implemented as computer programs where the parameter values are provided as input and where the random variables are drawn sequentially by making calls to a random number generator. The parameters $\theta$ govern the properties of interest of the generated data, whereas the random variables $V$ represent the stochastic variation inherent to the simulated process.

The mapping $M$ may be as complex as needed, and this generality of simulator-based models allows researchers to implement hypotheses about how the data were generated without having to make excessive compromises motivated by mathematical simplicity, or other reasons not related to the scientific question being investigated.

**Figure 1.**
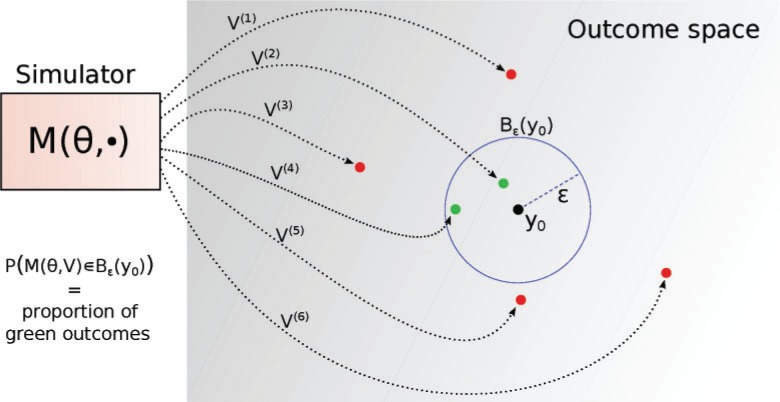
Illustration of the stochastic simulator $M$ run multiple times with a fixed value of $\theta$. The black dot ${{y}}_0$ is the observed data and the arrows point to different simulated data sets. Two outcomes, marked in green, are less than $\epsilon$ away from ${{y}}_0$. The proportion of such outcomes provides an approximation of the likelihood of $\theta$ for the observed data ${{y}}_0$.

Due to the presence of the random variables $V$, the outputs of the simulator fluctuate randomly even when using exactly the same values of the model parameters $\theta$. This means that we can consider the simulator to define a random variable $Y_\theta$ whose distribution is implicitly determined by the distribution of $V$ and the mapping $M$ acting on $V$ for a given $\theta$. For this reason, simulator-based models are sometimes called implicit models (Diggle and Gratton 1984). Using the properties of transformation of random variables, it is possible to formally write down the distribution of $Y_\theta$. For instance, for a fixed value of $\theta$, the probability that $Y_\theta$ takes values in an $\epsilon$ neighborhood $B_\epsilon({{y}}_0)$ around the observed data ${{y}}_0$ is equal to the probability to draw values of $V$ that are mapped to that neighborhood (Fig. 1),

(1) $$\mathrm{Pr}(Y_{\theta }\in B_{\epsilon }(y_{0}))=\mathrm{Pr}(M(\theta ,V)\in B_{\epsilon }(y_{0})).$$

Computing the probability analytically is impossible for complex models. But it is possible to test empirically whether a particular outcome $y_\theta$ of the simulation ends up in the neighborhood of ${{y}}_0$ or not (Fig. 1). We will see that this property of simulator-based models plays a key role in performing inference about their parameters.

### Example

As an example of a simulator-based model, we here present the simple yet analytically intractable model by Tanaka et al. (2006) for the spread of tuberculosis. We will use the model throughout the article for illustrating different concepts and methods.

**Figure 2.**
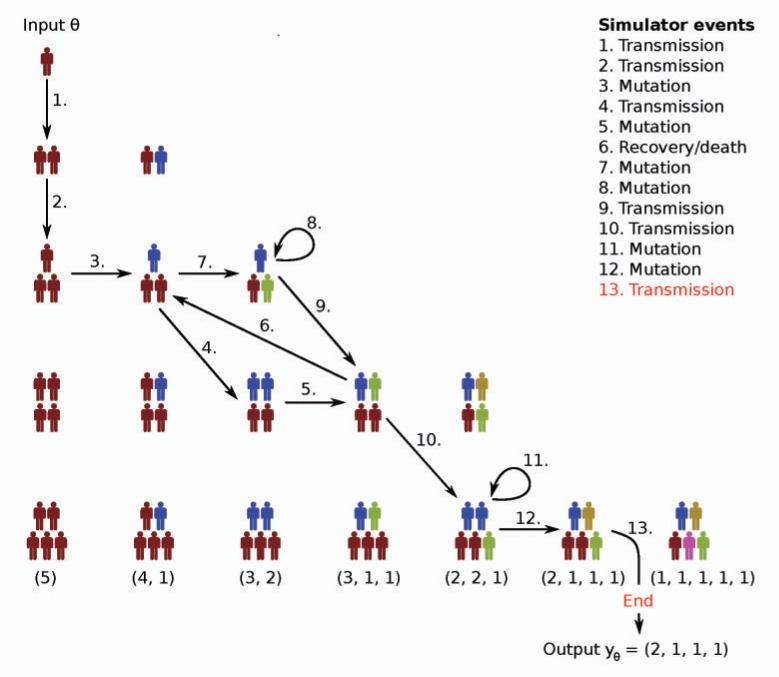
An example of a transmission process simulated under a parameter configuration $\theta$ without subsampling of the simulated infectious population. Arrows indicate the sequence of random events taking place in the simulation and different colors represent different haplotypes of the pathogen. The simulation starts with one infectious host who transmits the pathogen to another host. After one more transmission event, the pathogen undergoes a mutation within one of the three hosts infected so far (event three). As the sixth event in the simulation, one of the haplotypes is removed from the population due to the recovery/death of the corresponding host. The simulation stops when the infectious population size exceeds $m=5$ and the simulator outputs the generated $y_\theta$. The nodes not connected by arrows show all the other possible configurations of the infectious population, but which were not visited in this example run of the simulator. The bottom row lists the possible outputs of the simulator (cluster size vectors) under their corresponding population configuration.

The model begins with one infectious host and stops when a fixed number of infectious hosts $m$ is exceeded (Fig. 2). In the simulation, it is assumed that each infectious host randomly infects other individuals from an unlimited supply of hosts with the rate $\alpha$, each time transmitting a specific strain of the communicable pathogen, characterized by its haplotype. It is thus effectively assumed that a strong transmission bottleneck occurs, such that only a single strain is passed forward in each transmission event, despite the eventual genetic variation persisting in the within-host pathogen population. Further, each infected host is considered to be infectious immediately. The model states that a host stops being infectious, that is, recovers or dies, randomly with the rate $\delta$, and the pathogen of the host mutates randomly within the host at the rate $\tau$, thereby generating a novel haplotype under a single-locus infinite alleles model. The parameters of the model are thus $\theta=(\alpha,\delta,\tau)$. The output of the simulator is a vector of cluster sizes in the simulated population of infected hosts, where clusters are the groups of hosts infected by the same haplotype of the pathogen. After the simulation, a random sample of size $n < m$ is taken from the population yielding the vector of cluster sizes $y_\theta$ present in the sample. For example, $y_\theta = (6, 3, 2, 2, 1, 1, 1, 1, 1, 1, 1)$ corresponds to a sample of size $20$ containing one cluster with six infected hosts, one cluster with three hosts, two clusters with two hosts each, as well as seven singleton clusters. Note that this model of pathogen spread is atypical in the sense that the observation times of the infections are all left implicit in the sampling process, in contrast to the standard likelihood formulation used for infectious disease epidemiological models (Anderson and May 1992).

### Difficulties in Performing Statistical Inference

Values of the parameters $\theta$ that are plausible in the light of the observations ${{y}}_0$ can be determined via statistical inference either by finding values that maximize the probability in Equation (1) for some sufficiently small $\epsilon$ or by determining their posterior distribution. In more detail, in maximum likelihood estimation, the parameters are determined by maximizing the likelihood function $L(\theta)$,

(2) $$L(\theta )=\lim_{\epsilon \to 0}c_{\epsilon }\mathrm{Pr}(Y_{\theta }\in B_{\epsilon }(y_{0})),$$

where $c_{\epsilon}$ is a proportionality factor that may depend on $\epsilon$, which is needed when $ \Pr\big(Y_\theta \in B_\epsilon({{y}}_0)\big)$ shrinks to zero as $\epsilon$ approaches zero. If the output of the simulator can only take a countable number of values, $Y_\theta$ is called a discrete random variable and the definition of the likelihood simplifies to $L(\theta) = \Pr\big(Y_\theta = {{y}}_0\big)$, which equals the probability of simulating data equal to the observed data. In Bayesian inference, the essential characterization of the uncertainty about the model parameters is defined by their conditional distribution given the data, that is, the posterior distribution $p(\theta | {{y}}_0)$,

(3) $$p(\theta |y_{0})\propto L(\theta )p(\theta ),$$

where $p(\theta)$ is the prior distribution of the parameters.

For complex models neither the probability in Equation (1) nor the likelihood function $L(\theta)$ are available analytically in closed form as a function of $\theta$, which is the reason why statistical inference is difficult for simulator-based models.

**Figure 3.**
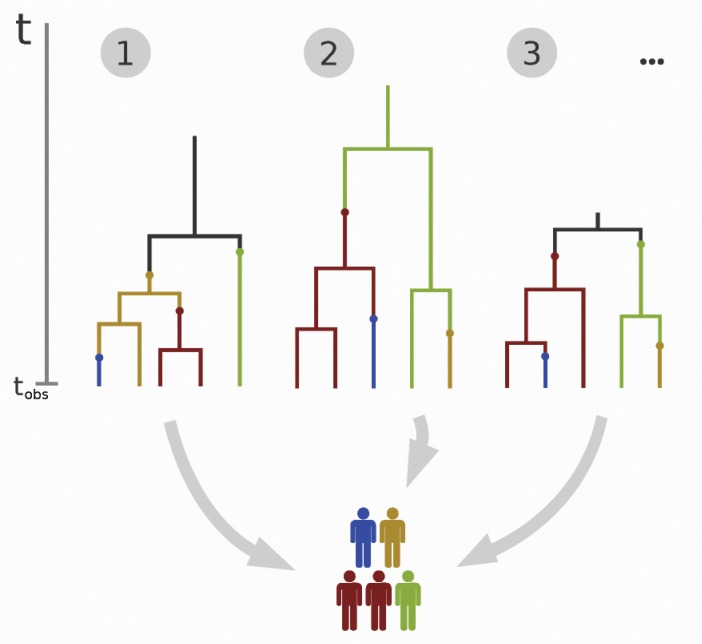
The transmission process in Figure 2 can also be described with transmission trees (Stadler 2011) paired with mutations. The trees are characterized by their structure, the length of their edges, and the mutations on the edges (marked with small circles that change the color of the edge, where colors represent the different haplotypes of the pathogen). The figure shows three examples of different trees that yield the same observed data at the observation time $t_{\text{obs}}$. Calculating the likelihood of a parameter value requires summing over all possible trees yielding the observed data, which is computationally impossible when the sample size is large.

For the model of tuberculosis transmission presented in the previous section, computing the likelihood function becomes intractable if the infectious population size $m$ is large, or if the death rate $\delta> 0$ (Stadler 2011). This is because for large $m$, the state space of the process, that is, the number of different cluster vectors, grows very quickly. This makes exact numerical calculation of the likelihood infeasible because in essence, every possible path to the outcome should be accounted for (Fig. 2). Moreover, if the death rate $\delta$ is nonzero, the process is allowed to return to previous states which further complicates the computations. Finally, the assumption that not all infectious hosts are observed contributes additionally to the intractability of the likelihood. Stadler (2011) approached the problem using transmission trees (Fig. 3). The likelihood function stays, however, intractable because of the vast number of different trees that all yield the same observed data and thus need to be considered when evaluating the likelihood of a parameter value.

### Inference via Rejection Sampling

We present here an algorithm for exact posterior inference that is applicable when $Y_\theta$ can only take countably many values, that is, if $Y_\theta$ is a discrete random variable. As shown above, in this case $L(\theta)=\Pr(Y_\theta={{y}}_0)$. The presented algorithm forms the basis of the algorithms for ABC discussed in the later sections.

In general, samples from the prior distribution $p(\theta)$ of the parameters can be converted into samples from the posterior $p(\theta | {{y}}_0)$ by retaining each sampled value with a probability proportional to $L(\theta)$. This can be done sequentially by first sampling a parameter value from the prior, $\theta \sim p(\theta)$ and then accepting the obtained value with the probability $L(\theta) / (\max_\theta L(\theta))$. This procedure corresponds to rejection sampling (see e.g., Robert and Casella 2004, Chapter 2). Now with the likelihood $L(\theta)$ being equal to the probability that $Y_\theta={{y}}_0$, the latter step can be implemented for simulator-based models even when $L(\theta)$ is not available analytically: we run the simulator and check whether the generated data equal the observed data. This gives the rejection algorithm for simulator-based models summarized as Algorithm 1. Rubin (1984) used it to provide intuition about how Bayesian inference about parameters works in general.


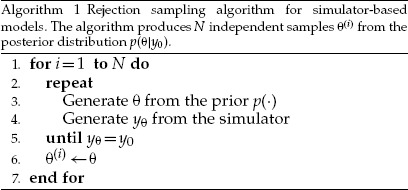


To obtain another interpretation of Algorithm 1, recall that for discrete random variables the posterior distribution $p(\theta | {{y}}_0)$ is, by definition, equal to the joint distribution of $\theta$ and $Y_\theta$, normalized by the probability that $Y_\theta={{y}}_0$. That is, the posterior is obtained by conditioning on the event $Y_\theta={{y}}_0$. We can thus understand the test for equality of $y_\theta$ and ${{y}}_0$ on line 5 of the algorithm as an implementation of the conditioning operation.

**Figure 4.**
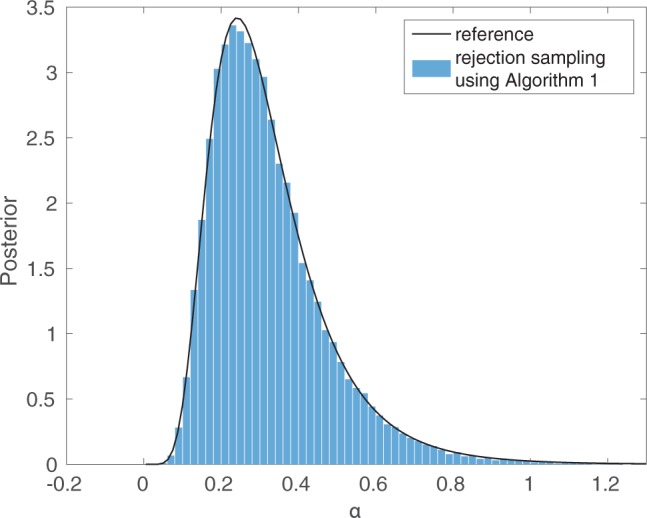
Exact inference for a simulator-based model of tuberculosis transmission. A very simple setting was chosen where the exact posterior can be numerically computed (black line), and where Algorithm 1 is applicable (blue bars).

To illustrate Algorithm 1, we generated a synthetic data set ${{y}}_0$ from the tuberculosis transmission model by running the simulator with the parameter values $\alpha=0.2$, $\delta=0$, $\tau = 0.198$, and setting the population size to $m=20$. We further assumed that the whole population is observed, which yielded the observed data ${{y}}_0 = (6, 3, 2, 2, 1, 1, 1, 1, 1, 1, 1)$. The assumptions about the size of the population, and that the whole population was observed, are unrealistic but they enable a comparison to the exact posterior distribution, which in this setting can be numerically computed using Theorem 1 of Stadler (2011). In this case, the histogram of samples obtained with Algorithm 1 matches the posterior distribution very accurately (Fig. 4). To obtain this result, we assumed that both of the parameters $\delta$ and $\tau$ were known and assigned a uniform prior distribution in the interval $(0.005,2)$ for the sole unknown parameter, the transmission rate $\alpha$. A total of 20 million data sets $y_\theta$ were simulated, out of which 40,000 matched ${{y}}_0$ (acceptance rate of 0.2%).

## Fundamentals of approximate Bayesian computation

### The Rejection ABC Algorithm

While Algorithm 1 produces independent samples from the posterior, the probability that the simulated data equal the observed data is often negligibly small, which renders the algorithm impractical as virtually no simulated realizations of $\theta$ will be accepted. The same problem holds true if the generated data can take uncountably many values, that is, when $Y_\theta$ is a continuous random variable.

To make inference feasible, the acceptance criterion $y_\theta={{y}}_0$ in Algorithm 1 can be relaxed to

(4) $$d(y_{\theta },y_{0})\leq \epsilon ,$$

where $\epsilon >0$ and $d(y_\theta,{{y}}_0) \ge 0$ is a “distance” function that measures the discrepancy between the two data sets, as considered relevant for the inference. With this modification, Algorithm 1 becomes the rejection ABC algorithm summarized as Algorithm 2. The first implementation of this algorithm appeared in the work by Pritchard et al. (1999).


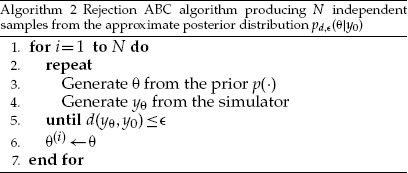


Algorithm 2 does not produce samples from the posterior $p(\theta | {{y}}_0)$ in Equation (3) but samples from an approximation $p(\theta | {{y}}_0)abc$,

(5) $$p_{d,\epsilon }(\theta |y_{0})abc\propto \mathrm{Pr}(d(Y_{\theta },y_{0})\leq \epsilon )p(\theta ),$$

which is the posterior distribution of $\theta$ conditional on the event $d(Y_\theta,{{y}}_0) \le \epsilon$. Equation (5) is obtained by approximating the intractable likelihood function $L(\theta)$ in Equation (2) with $L(\theta)abc$,

(6) $$L_{d,\epsilon }(\theta )abc\propto \mathrm{Pr}(d(Y_{\theta },y_{0})\leq \epsilon ).$$

The approximation is two-fold. First, the distance function $d$ is generally not a metric in the mathematical sense, namely $d(y_\theta,{{y}}_0)=0$ even if $y_\theta \neq {{y}}_0$. This may happen, for example, when $d$ is defined through summary statistics that remove information from the data (see below). Second, $\epsilon$ is chosen large enough so that enough samples will be accepted. Intuitively, the likelihood of a parameter value is approximated by the probability that running the simulator with said parameter value produces data within $\epsilon$ distance of ${{y}}_0$ (Fig. 1).

The distance $d$ is typically computed by first reducing the data to suitable summary statistics $t = T(y)$ and then computing the distance $d_T$ between them, so that $d(y_\theta,{{y}}_0)=d_T(t,{{t}}_0)$, where $d_T$ is often the Euclidean or some other metric for the summary statistics. When combining different summary statistics, they are usually re-scaled so that they contribute equally to the distance (as, e.g., done by Pritchard et al. 1999).

In addition to the accuracy of the approximation $p(\theta | {{y}}_0)abc$, the distance $d$ and the threshold $\epsilon$ also influence the computing time required to obtain samples. For instance, if $\epsilon=0$ and the distance $d$ is such that $d(y,{{y}}_0) = 0$ if and only if $y={{y}}_0$, then Algorithm 2 becomes Algorithm 1 and $p(\theta | {{y}}_0)abc$ becomes $p(\theta | {{y}}_0)$ but the computing time to obtain samples from $p(\theta | {{y}}_0)abc$ would typically be impractically large. Hence, on a very fundamental level, there is a trade-off between statistical and computational efficiency in ABC (see e.g., Beaumont et al. 2002, p. 2027).

We next illustrate Algorithm 2 and the mentioned trade-off using the previous example about tuberculosis transmission. Two distances $d_1$ and $d_2$ are considered,

(7) $$\begin{array}{c} d_{1}(y_{\theta },y_{0})=|T_{1}(y_{\theta })-T_{1}(y_{0})|,\;d_{2}(y_{\theta },y_{0})=|T_{2}(y_{\theta })-T_{2}(y_{0})|, \end{array}$$

where $T_1$ is the number of clusters contained in the data divided by the sample size $n$ and $T_2$ is a genetic diversity measure defined as $T_2(y) = 1 - \sum_i (n_i/n)^2$, where $n_i$ is the size of the $i$-th cluster. For ${{y}}_0 = (6, 3, 2, 2, 1, 1, 1, 1, 1, 1, 1)$, we have $T_1({{y}}_0)=11/20=0.55$ and $T_2({{y}}_0) = 0.85$. For both $d_1$ and $d_2$, the absolute difference between the summary statistics is used as the metric $d_T$.

**Figure 5.**
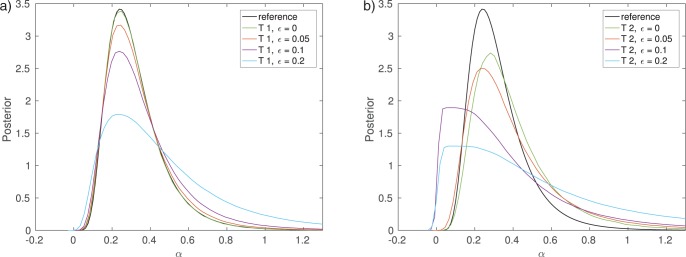
Inference results for the transmission rate $\alpha$ of tuberculosis. The plots show the posterior distributions obtained with Algorithm 2 and 20 million simulated data sets (proposals). a) Cluster frequency as a summary statistic. b) Genetic diversity as a summary statistic.

For this example, using the summary statistic $T_1$ instead of the full data does not lead to a visible deterioration of the inferred posterior when $\epsilon = 0$ (Fig. 5a). For summary statistic $T_2$, however, there is a clear difference as the posterior mode and mean are shifted to larger values of $\alpha$ and the posterior variance is larger too (Fig. 5b). In both cases, increasing $\epsilon$, that is, accepting more parameters, leads to an approximate posterior distribution that is less concentrated than the true posterior.

**Figure 6.**
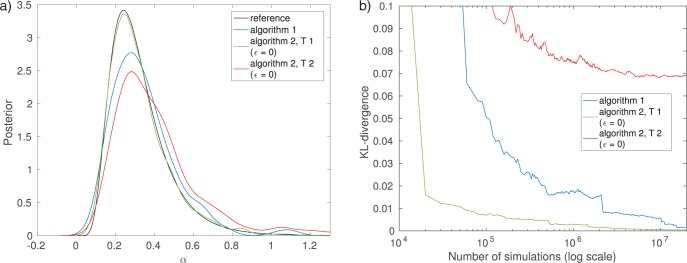
Comparison of the efficiency of Algorithms 1 and 2. Smaller KL divergence means more accurate inference of the posterior distribution. Note that the stopping criterion of the algorithm has here been changed to be the total number of runs of the simulator instead of the number of accepted samples. a) Results after 100,000 simulations. b) Accuracy versus computational cost.

Algorithm 2 with summary statistic $T_1$ produces results comparable to Algorithm 1 but from the computational efficiency point of view the number of simulations required to obtain the approximate posterior differs between the two algorithms. It can be seen that for a computational budget of 100,000 simulations, the posterior obtained by Algorithm 1 differs substantially from the exact posterior, while the posterior from Algorithm 2 with $T_1$ is still matching it well (Fig. 6a). The relatively poor result with Algorithm 1 is due to its low acceptance rate (here 0.2%). While the accepted samples do follow the exact posterior $p(\theta | {{y}}_0)$, the algorithm did not manage to produce enough accepted realizations within the computational budget available, which implies that the Monte Carlo error of the posterior approximation remains nonnegligible.

Plotting the number of data sets simulated versus the accuracy of the inferred posterior distribution allows us to further study the trade-off between statistical and computational efficiency between the different algorithms (Fig. 6b). The accuracy is measured by the Kullback–Leibler (KL) divergence (Kullback and Leibler 1951) between the exact and the inferred posterior. Algorithm 2 with summary statistic $T_1$ features the best trade-off, whereas using summary statistic $T_2$ performs the worst. The curve of the latter one flattens out after approximately 1 million simulations, showing the approximation error introduced by using the summary statistic $T_2$. For Algorithm 1, nonzero values of the KL divergence are due to the Monte Carlo error only and it will approach the true posterior as the number of simulations grows. When using summary statistics, nonzero values of the KL divergence are due to both the Monte Carlo error and the use of the summary statistics. In this particular example, the error caused by the summary statistic $T_1$ is, however, negligible.

### Choice of the Summary Statistics

If the distance $d$ is computed by projecting the data to summary statistics followed by their comparison using a metric in the summary statistics space (e.g., the Euclidean distance), the quality of the inference hinges on the summary statistics chosen (Figs. 5 and 6).

For consistent performance of ABC algorithms, the summary statistics should be sufficient for the parameters, but this is often not the case. Additionally, with the increase in the number of summary statistics used, more simulations tend to be rejected so that an increasing number of simulation runs is needed to obtain a satisfactory number of accepted parameter values, making the algorithm computationally extremely inefficient. This is known as the curse of dimensionality for ABC (see also the discussion in the review paper by Beaumont 2010).

One of the main remedies to the above issue is to efficiently choose informative summary statistics. Importantly, the summary statistics that are informative for the parameters in a neighborhood of the true parameter value, and the summary statistics most informative globally, are significantly different (Nunes and Balding 2010). General intuition suggests that the set of summary statistics that are locally sufficient would be a subset of the globally sufficient ones. Therefore, a good strategy seems to first find a locality containing the true parameter with high enough probability and then choose informative statistics depending on that locality. However, this can be difficult in practice because rather different parameter values can produce summary statistics that are contained in the same locality.

In line with the above, Nunes and Balding (2010), Fearnhead and Prangle (2012), and Aeschbacher et al. (2012) first defined “locality” through a pilot ABC run and then chose the statistics in that locality. Four methods for choosing the statistics are generally used: (i) a sequential scheme based on the principle of approximate sufficiency (Joyce and Marjoram 2008); (ii) selection of a subset of the summary statistics maximizing prespecified criteria such as the Akaike information criterion (used by Blum et al. 2013) or the entropy of a distribution (used by Nunes and Balding 2010); (iii) partial least square regression which finds linear combinations of the original summary statistics that are maximally decorrelated and at the same time highly correlated with the parameters (Wegmann et al. 2009); (iv) assuming a statistical model between parameters and transformed statistics of simulated data, summary statistics are chosen by minimizing a loss function (Aeschbacher et al. 2012; Fearnhead and Prangle 2012). For comparison of the above methods in simulated and practical examples, we refer readers to the work by Blum and François (2010), Aeschbacher et al. (2012), and Blum et al. (2013).

### Choice of the Threshold

Having the distance function $d$ specified, possibly using summary statistics, the remaining factor in the approximation of the posterior in Equation (5) is the specification of the threshold $\epsilon$.

**Figure 7.**
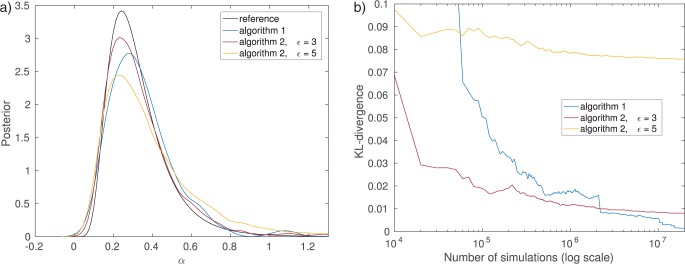
Comparison of the trade-off between Monte Carlo error and bias. Algorithm 1 is equivalent here to Algorithm 2 with $\epsilon=0$. Smaller KL divergences mean more accurate inference of the posterior distribution. a) Results after 100,000 simulations. b) Accuracy versus computational cost.

Larger values of $\epsilon$ result in biased approximations $p(\theta | {{y}}_0)abc$ (see e.g., Fig. 5). The gain is a faster algorithm, meaning a reduced Monte Carlo error as one is able to produce more samples per unit of time. Therefore, when specifying the threshold the goal is to find a good balance between the bias and the Monte Carlo error. We illustrate this using Algorithm 2 with the full data without reduction to summary statistics [in other words, $T(y)=y$]. In this case, Algorithm 2 with $\epsilon=0$ is identical to Algorithm 1. Here the choice $\epsilon=3$ results in a better posterior compared to $\epsilon=0$ when using a maximal number of 100,000 simulations (Fig. 7a). This means that the gain from reduced Monte Carlo error is greater than the loss incurred by the bias. But this is no longer true for $\epsilon=5$ where the bias dominates. Eventually, the exact method will converge to the true posterior, whereas the other two continue to suffer from the bias caused by the larger threshold (Fig. 7b). However, with smaller computational budgets (less than 2 million simulations in our example), more accurate results are obtained with the nonzero threshold $\epsilon=3$.

The choice of the threshold is typically made by experimenting with a precomputed pool of simulation–parameter pairs $(y_\theta, \theta)$. Rather than setting the threshold value by hand, it is often determined by accepting some small proportion of the simulations (e.g., 1%, see Beaumont et al. 2002). The choice between different options can be made more rigorous by using some of the simulated data sets in the role of the observed data and solving the inference problem for them using the remaining data sets. As the data-generating parameters are known for the simulated observations, different criteria, such as the mean squared error (MSE) between the mean of the approximation and the generating parameters can be used to make the choice [see e.g. Faisal et al. (2013) and the section on validation of ABC]. This also allows one to assess the reliability of the inference procedure. Prangle et al. (2014) discuss the use of the coverage property (Wegmann et al. 2009) as the criterion to choose the threshold value $\epsilon$. Intuitively, the coverage property tests if the parameter values $\theta^*$ used to artificially generate a data set ${{y}}_0^*$ are covered by the credible intervals constructed from the ABC output for ${{y}}_0^*$ at correct rates (i.e., $\alpha\%$ credible intervals should contain the true parameter in $\alpha\%$ of the tests).

If one plans to increase the computational budget after initial experiments, theoretical convergence results can be used to adjust the threshold value. Barber et al. (2015) provide convergence results for an optimal $\epsilon$ sequence with respect to the MSE of a posterior expectation (e.g., the posterior mean). The theoretically optimal sequence for the threshold $\epsilon$ is achieved by making it proportional to $N^{-1/4}$ as $N\to \infty$, where $N$ is the number of accepted samples. If the constant in this relation is estimated in a pilot run, one can compute the new theoretically optimal threshold based on the planned increase in the computational budget. Blum (2010) derives corresponding results using an approach based on conditional density estimation, finding that $\epsilon$ should optimally be proportional to $N_{s}^{-1/(d+5)}$ as $N_s\to\infty$, where $d$ is the dimension of the parameter space and $N_s$ the total number of simulations performed [see also Fearnhead and Prangle (2012), Silk et al. (2013), and Biau et al. (2015), for similar results].

## Beyond simple rejection sampling

The basic rejection ABC algorithm is essentially a trial and error scheme where the trial (proposal) values are sampled from the prior. We now review three popular algorithms that seek to improve upon the basic rejection approach. The first two aim at constructing proposal distributions that are closer to the posterior, whereas the third is a correction method that aims at adjusting samples obtained by ABC algorithms so that they are closer to the posterior.

### Markov Chain Monte Carlo ABC

The Markov chain Monte Carlo (MCMC) ABC algorithm is based on the Metropolis–Hastings MCMC algorithm that is often used in Bayesian statistics (Robert and Casella 2004, Chapter 7). In order to leverage this algorithm, we write $p(\theta | {{y}}_0)abc$ in Equation (5) as the marginal distribution of $p_{d,\epsilon}(\theta,y | {{y}}_0)$,

(8) $$p_{d,\epsilon }(\theta ,y|y_{0})\propto p(\theta )p(y|\theta )1[d(y,y_{0})\leq \epsilon ],$$

where $p(y|\theta)$ denotes the probability density (mass) function of $Y_\theta$, and ${\mathbb{1}}[d(y,{{y}}_0)\le \epsilon]$ equals one if $d(y,{{y}}_0)\le \epsilon$ and zero otherwise. Importantly, while $p(y|\theta)$ is generally unknown for simulator-based models, it is still possible to use $p_{d,\epsilon}(\theta,y |{{y}}_0)$ as the target distribution in a Metropolis–Hastings MCMC algorithm by choosing the proposal distribution in the right way. The obtained (marginal) samples of $\theta$ then follow the approximate posterior $p(\theta | {{y}}_0)abc$.

Assuming that the Markov chain is at iteration $i$ in state ${x}^{(i)}=({\theta}^{(i)},{y}^{(i)})$ where $d({y}^{(i)},{{y}}_0)\le \epsilon$, the Metropolis–Hastings algorithm involves sampling candidate states $x=(\theta,y)$ from a proposal distribution $q(x| {x}^{(i)})$ and accepting the candidates with the probability $A(x|{x}^{(i)})$,

(9) $$A(x|x^{\left(i\right)})=\min\left(1,\frac{p_{d,\epsilon }(x|y_{0})q(x^{\left(i\right)}|x)}{p_{d,\epsilon }(x^{\left(i\right)}|y_{0})q(x|x^{\left(i\right)})}\right).$$

Choosing the proposal distribution such that the move from ${x}^{(i)}=({\theta}^{(i)},{y}^{(i)})$ to $x=(\theta,y)$ does not depend on the value of ${y}^{(i)}$, and that $y$ is sampled from the simulator-based model with parameter value $\theta$ irrespective of ${\theta}^{(i)}$, we have

(10) $$q(x|x^{\left(i\right)})=q(\theta |\theta^{\left(i\right)})p(y|\theta ),$$

where $q(\theta|{\theta}^{(i)})$ is a suitable proposal distribution for $\theta$. As a result of this choice, the unknown quantities in Equation (9) cancel out,

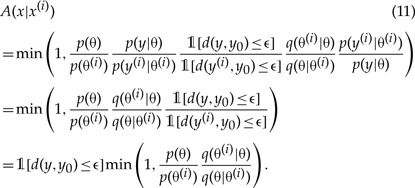


This means that the acceptance probability is only probabilistic in $\theta$ since a proposal $(\theta,y)$ is immediately rejected if the condition $d(y,{{y}}_0)\le \epsilon$ is not met. While the Markov chain operates in the $(\theta,y)$ space, the choice of the proposal distribution decouples the acceptance criterion into an ordinary Metropolis–Hastings criterion for $\theta$ and the previously seen ABC rejection criterion for $y$. The resulting algorithm, shown in full in the Appendix, is known as MCMC ABC algorithm and was introduced by Marjoram et al. (2003).

An advantage of the MCMC ABC algorithm is that the parameter values do not need to be drawn from the prior, which most often hampers the rejection sampler by incurring a high rejection rate of the proposals. As the Markov chain converges, the proposed parameter values follow the posterior with some added noise. A potential disadvantage, however, is the continuing presence of the rejection condition $d(y,{{y}}_0)\le \epsilon$ which dominates the acceptance rate of the algorithm. Parameters in the tails of the posteriors have, by definition, a small probability to generate data $y_\theta$ satisfying the rejection condition, which can lead to a “sticky” Markov chain where the state tends to remain constant for many iterations.

### Sequential Monte Carlo ABC

The sequential Monte Carlo (SMC) ABC algorithm can be considered as an adaptation of importance sampling which is a popular technique in statistics (see e.g., Robert and Casella 2004, Chapter 3). If one uses a general distribution $\phi(\theta)$ in place of the prior $p(\theta)$, Algorithm 2 produces samples that follow a distribution proportional to $\phi(\theta) \Pr(d(Y_\theta,{{y}}_0)\le \epsilon)$. However, by weighting the accepted parameters ${\theta}^{(i)}$ with ${{w}}^{(i)}$,

(12) $$w^{\left(i\right)}\propto \frac{p(\theta^{\left(i\right)})}{\phi (\theta^{\left(i\right)})},$$

the resulting weighted samples follow $p(\theta | {{y}}_0)abc$. This kind of trick is used in importance sampling and can be employed in ABC to iteratively morph the prior into a posterior.

**Figure 8.**
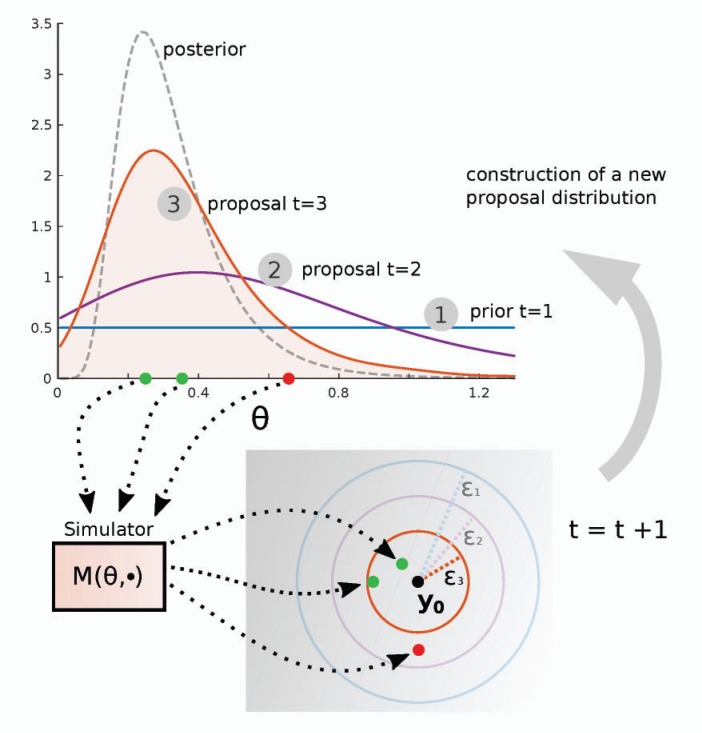
Illustration of sequential Monte Carlo ABC using the tuberculosis example. The first proposal distribution is the prior and the threshold value used is $\epsilon_1$. The proposal distribution in iteration $t$ is based on the sample of size $N$ from the previous iteration. The threshold value $\epsilon_t$ is decreased at every iteration as the proposal distributions become similar to the true posterior. The figure shows parameters drawn from the proposal distribution of the third iteration ($t=3$). The red proposal is rejected because the corresponding simulation outcome is too far from the observed data $y_0$. At iteration $t=2$, however, it would have been accepted. After iteration $t$, the accepted parameter values follow the approximate posterior ${p_{d,\epsilon_t}}(\theta | {{y}}_0)$. As long as the threshold values $\epsilon_t$ decrease, the approximation becomes more accurate at each iteration.

The basic idea is to use a sequence of shrinking thresholds $\epsilon_t$ and to define the proposal distribution $\phi_t$ at iteration $t$ based on the weighted samples ${\theta}^{(i)}_{t-1}$ from the previous iteration (Fig. 8). This is typically done by defining a mixture distribution,

(13) $$\phi_{t}(\theta )=\frac{1}{N}\sum_{i=1}^{N}q_{t}(\theta |\theta_{t-1}^{\left(i\right)})w_{t-1}^{\left(i\right)},$$

where $q_t(\theta|{\theta}^{(i)}_{t-1})$ is often a Gaussian distribution with mean ${\theta}^{(i)}_{t-1}$ and a covariance matrix estimated from the samples. Sampling from $\phi_t$ can be done by choosing ${\theta}^{(i)}_{t-1}$ with probability ${{w}}^{(i)}_{t-1}$ and then perturbing the chosen parameter according to $q_t$. The proposed sample is then accepted or rejected as in Algorithm 2 and the weights of the accepted samples are computed with Equation (12). Such iterative algorithms were proposed by Sisson et al. (2007); Beaumont et al. (2009); Toni et al. (2009) and are called SMC ABC algorithms or population Monte Carlo ABC algorithms. The algorithm by Beaumont et al. (2009) is given in the Appendix.

Similar to the MCMC ABC, the samples proposed by the SMC algorithm follow the posterior ${p_{d,\epsilon_t}}(\theta | {{y}}_0)$ with some added noise. The proposed parameter values are drawn from the prior only at the first iteration after which adaptive proposal distributions $\phi_t$ closer to the true posterior are used (see Fig. 8 for an illustration). This reduces the running time as the number of rejections is lower compared to the basic rejection ABC algorithm. For small values of $\epsilon$, however, the probability to accept a parameter value becomes very small, even if the parameter value was sampled from the true posterior. This results in long computing times in the final iterations of the algorithm without notable improvements in the approximation of the posterior.

### Post-Sampling Correction Methods

We assume here that the distance $d(y_\theta,{{y}}_0)$ is specified in terms of summary statistics, that is, $d(y_\theta,{{y}}_0) = d_T(t_\theta,{{t}}_0)$, with $t_\theta = T(y_\theta)$ and ${{t}}_0=T({{y}}_0)$. As $\epsilon$ decreases to zero, the approximate posterior $p(\theta | {{y}}_0)abc$ in Equation (5) converges to $p(\theta|{{t}}_0)$, where we use $p(\theta|t)$ to denote the conditional distribution of $\theta$ given a value of the summary statistics $t$. While small values of $\epsilon$ are thus preferred in theory, making them too small is not feasible in practice because of the correspondingly small acceptance rate and the resulting large Monte Carlo error. We here present two schemes that aim at adjusting $p(\theta | {{y}}_0)abc$ without further sampling so that the adjusted distribution is closer to $p(\theta|{{t}}_0)$.

For the first scheme, we note that if we had a mechanism to sample from $p(\theta|t)$, we could sample from the limiting approximate posterior by using $t={{t}}_0$. The post-sampling correction methods in the first scheme thus estimate $p(\theta|t)$ and use the estimated conditional distributions to sample from $p(\theta|{{t}}_0)$. In order to facilitate sampling, $p(\theta|t)$ is expressed in terms of a generative (regression) model,

(14) θ=f(t,ξ),

where $f$ is a vector-valued function and $\xi$ a vector of random variables for the residuals. By suitably defining $f$, we can assume that the random variables of the vector $\xi$ are independent, of zero mean and equal variance, and that their distribution $p_\xi$ does not depend on $t$. Importantly, the model does not need to hold for all $t$ because, ultimately, we would like to sample from it using $t={{t}}_0$ only. Assuming that the model $f$ holds for $d_T(t,{{t}}_0)\le \delta$ and that we have (weighted) samples $({{t}}^{(i)},{{\tilde{\theta}}}^{(i)})=(T({{y_\theta}}^{(i)}),{{\tilde{\theta}}}^{(i)})$ available from an ABC algorithm with a threshold $\epsilon\le\delta$, the model $f$ can be estimated by regressing $\theta$ on the summary statistics $t$.

In order to sample $\theta$ using the estimated model $\hat{f}$, we need to know the distribution of $\xi$. For that, the residuals ${{\xi}}^{(i)}$ are determined by solving the regression equation,

(15) $${\tilde{\theta }}^{\left(i\right)}=\hat{f}(t^{\left(i\right)},\xi^{\left(i\right)}).$$

The residuals ${{\xi}}^{(i)}$ can be used to estimate $p_\xi$, or as usually is the case in ABC, be directly employed in the sampling of the $\theta$,

(16) $$\theta^{\left(i\right)}=\hat{f}(t_{0},\xi^{\left(i\right)}).$$

If the original samples $({{t}}^{(i)},{{\tilde{\theta}}}^{(i)})$ are weighted, both the ${{\xi}}^{(i)}$ and the new “adjusted” samples ${\theta}^{(i)}$ inherit the weights. By construction, if the relation between $t$ and $\theta$ is estimated correctly, the (weighted) samples ${\theta}^{(i)}$ follow $p(\theta | {{y}}_0)abc$ with $\epsilon=0$.

**Figure 9.**
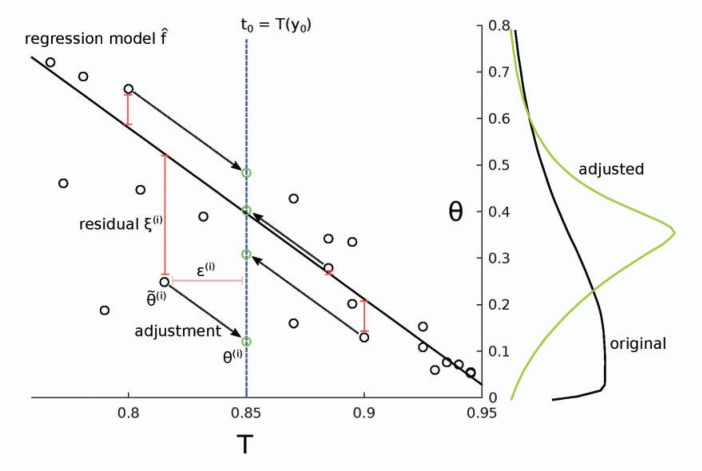
Illustration of the linear regression adjustment (Beaumont et al. 2002). First, the regression model $\hat{f}$ is learned and then, based on $\hat{f}$, the simulations are adjusted as if they were sampled from $p(\theta | {{y}}_0)abc$ with $\epsilon=0$. Note that the residuals ${{\xi}}^{(i)}$ are preserved. The change in the posterior densities after the adjustment is shown on the right. Here, the black (original) and green (adjusted) curves are the same as in Figure 10(b).

**Figure 10.**
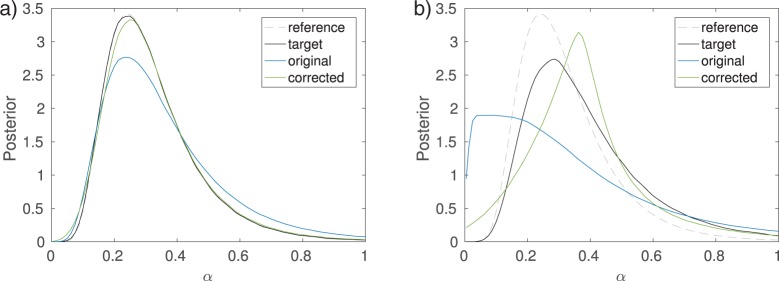
Linear regression adjustment (Beaumont et al. 2002). applied to the example model of the spread of tuberculosis (compare to Fig. 5). The target distribution of the adjustment is the posterior $p(\theta | {{y}}_0)abc$ with the threshold decreased to $\epsilon=0$. Note that when using summary statistic $T_2$ the target distribution is substantially different from the true posterior (reference) because of the bias incurred by $T_2$. a) $T_1$ with $\epsilon=0.1$. b) $T_2$ with $\epsilon=0.1$.

In most models $f$ employed so far, the individual components of $\theta$ are treated separately, thus not accounting for possible correlations between them. For this paragraph we thus let $\theta$ be a scalar. The first regression model used was linear (Beaumont et al. 2002),

(17) $$\begin{array}{ccc} \theta & \;=f_{1}(t,\xi ), & f_{1}(t,\xi )=\alpha +(t-t_{0}{)}^{⊤}\beta +\xi , \end{array}$$

which results in the adjustment ${\theta}^{(i)} = {{\tilde{\theta}}}^{(i)} - ({{t}}^{(i)}-{{t}}_0)^\top\hat{\beta}$, where $\hat{\beta}$ is the learned regression coefficient (Fig. 9). When applied to the model of the spread of tuberculosis, with summary statistic $T_1$ [see Equation (7)], the adjustment is able to correct the bias caused by the nonzero threshold $\epsilon=0.1$, that is the estimated model $\hat{f}$ is accurate (Fig. 10a). With summary statistic $T_2$, the threshold $\epsilon=0.1$ is too large for accurate adjustment, although the result is still closer to the target distribution than the original (Figure 10b). Note also that here the target distribution of the adjustment is substantially different from the true posterior due to the bias incurred by summary statistic $T_2$.

Also nonlinear models $f$ have been proposed. Blum (2010) assumed a quadratic model,

(18) $$\begin{array}{ccc} \theta & \;=f_{2}(t,\xi ), & f_{2}(t,\xi )=\alpha +(t-t_{0}{)}^{⊤}\beta +\frac{1}{2}(t-t_{0}{)}^{⊤}\gamma (t-t_{0})+\xi , \end{array}$$

where $\gamma$ is a symmetric matrix that adds a quadratic term to the linear adjustment. A more general nonlinear model was considered by Blum and François (2010),

(19) $$\begin{array}{cccc} \theta & \;=f_{3}(t,\xi ), & f_{3}(t,\xi ) & \;=m(t)+\sigma (t)\xi , \end{array}$$

where $m(t)$ models the conditional mean and $\sigma(t)$ the conditional standard deviation of $\theta$. Both functions were fitted using a multi-layer neural network, and denoting the learned functions by $\hat{m}$ and $\hat{\sigma}$, the following adjustments were obtained

(20) $$\theta^{\left(i\right)}=\hat{m}(t_{0})+\hat{\sigma }(t_{0})\hat{\sigma }(t^{\left(i\right)}{)}^{-1}({\tilde{\theta }}^{\left(i\right)}-\hat{m}(t^{\left(i\right)})).$$

The term $\hat{m}({{t}}_0)$ is an estimate of the posterior mean of $\theta$, whereas $\hat{\sigma}({{t}}_0)$ is an estimate of the posterior standard deviation of the parameter. They can both be used to succinctly summarize the posterior distribution of $\theta$.

A more complicated model $f(t,\xi)$ is not necessarily better than a simpler one. It depends on the amount of training data available to fit it, that is, the amount of original samples $({{t}}^{(i)},{{\tilde{\theta}}}^{(i)})$ that satisfy $d_T(t,{{t}}_0)\le \delta$. The different models presented above were compared by Blum and François (2010) who also pointed out that techniques for model selection from the regression literature can be used to select among them.

While the first scheme to adjust $p(\theta | {{y}}_0)abc$ consists of estimating $p(\theta|t)$, the second scheme consists of estimating $p( t | \theta)$, that is the conditional distribution of the summary statistics given a parameter value. The rationale of this approach is that knowing $p(t | \theta)$ implies knowing the approximate likelihood function $L(\theta)abc$ for $\epsilon \to 0$, because $p({{t}}_0 |\theta) = \lim_{\epsilon \to 0} L(\theta)abc$ when the distance $d(y_\theta,{{y}}_0)$ is specified in terms of summary statistics.

Importantly, $p(t|\theta)$ does not need to be known everywhere but only locally around ${{t}}_0$, where $d_T(t,{{t}}_0) \le \epsilon$.

If we use $p_{\epsilon}(t|\theta)$ to denote the distribution of $t$ conditional on $\theta$ and $d_T(t,{{t}}_0) \le \epsilon$, Leuenberger and Wegmann (2010) showed that $p_\epsilon({{t}}_0|\theta)$ takes the role of a local likelihood function and $p(\theta | {{y}}_0)abc$ the role of a local prior, and that the local posterior equals the true posterior $p(\theta |{{t}}_0)$.

The functional form of $p_{\epsilon}(t|\theta)$ is generally not known. However, as in the first scheme, running an ABC algorithm with threshold $\epsilon$ provides data $({{t}}^{(i)},{{\tilde{\theta}}}^{(i)})$ that can be used to estimate a model of $p_{\epsilon}(t|\theta)$. Since the model does not need to hold for all values of the summary statistics, but only for those in the neighborhood of ${{t}}_0$, Leuenberger and Wegmann (2010) proposed to model $p_{\epsilon}(t|\theta)$ as Gaussian with constant covariance matrix and a mean depending linearly on $\theta$. When the samples $({{t}}^{(i)},{{\tilde{\theta}}}^{(i)})$ are used to approximate $p(\theta | {{y}}_0)abc$ as a kernel density estimate, the Gaussianity assumption on $p_{\epsilon}(t|\theta)$ facilitates the derivation of closed-form formulae to adjust the kernel density representation of $p(\theta | {{y}}_0)abc$ so that it becomes an approximation of $p(\theta|{{t}}_0)$ (Leuenberger and Wegmann 2010).

While Leuenberger and Wegmann (2010) modeled $p_{\epsilon}(t|\theta)$ as Gaussian, other models can be used as well. Alternatively, one may make the mean of the Gaussian depend nonlinearly on $\theta$ and allow the covariance of the summary statistic depend on $\theta$. This was done by Wood (2010) and the model was found rich enough to represent $p(t|\theta)$ for all values of the summary statistics and not only for those in the neighborhood of the observed one.

## Recent developments

We here present recent advances that aim to make ABC both computationally and statistically more efficient. This presentation focuses on our own work (Gutmann et al. 2014; Gutmann and Corander 2016).

### Computational Efficiency

The computational cost of ABC can be attributed to two main factors:

(1) Most of the parameter values result in large distances between the simulated and observed data and those parameter values for which the distances tend to be small are unknown.
(2) Generating simulated data sets, that is, running the simulator, may be costly.

**Figure 11.**
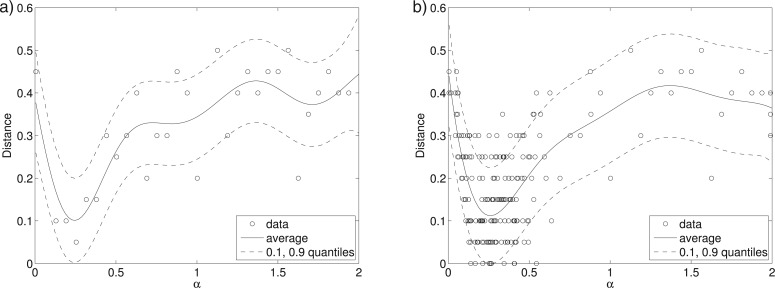
The basic idea of BOLFI is to model the distance, and to prioritize regions of the parameter space where the distance tends to be small. The solid curves show the modeled average behavior of the distance $d_1(Y_\theta,{{y}}_0)$, and the dashed curves its variability for the tuberculosis example. a) After initialization (30 data points). b) After active data acquisition (200 data points).

**Figure 12.**
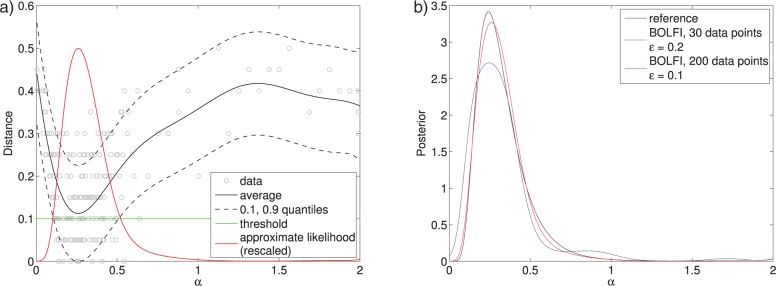
In BOLFI, the estimated model of $d(y_\theta,{{y}}_0)$ is used to approximate $L(\theta)abc$ by computing the probability that the distance is below a threshold $\epsilon$. This kind of likelihood approximation leads to a model-based approximation of $p(\theta | {{y}}_0)abc$. The KL-divergence between the reference solution and the BOLFI solution with 30 data points is 0.09, and for 200 data points it is 0.01. Comparison with Figure 6 shows that BOLFI increases the computational efficiency of ABC by several orders of magnitude. a) Approximate likelihood function. b) Model-based posteriors.

MCMC ABC and SMC ABC were partly introduced to avoid proposing parameters in regions where the distance is large. Nonetheless, typically millions of simulations are needed to infer the posterior distribution of a handful of parameters only. A key obstacle to efficiency in these algorithms is the continued presence of the rejection mechanism $d(y_\theta,{{y}}_0)\le \epsilon$, or more generally, the online decisions about the similarity between $y_\theta$ and ${{y}}_0$. In recent work, Gutmann and Corander (2016) proposed a framework called Bayesian optimization for likelihood-free inference (BOLFI) for performing ABC which overcomes this obstacle by learning a probabilistic model about the stochastic relation between the parameter values and the distance (Fig. 11). After learning, the model can be used to approximate $L(\theta)abc$, and thus $p(\theta | {{y}}_0)abc$, for any $\epsilon$ without requiring further runs of the simulator (Fig. 12).

Like the post-sampling correction methods presented in the previous section, BOLFI relies on a probabilistic model to make ABC more efficient. However, the quantities modeled differ, since in the post-sampling correction methods the relation between summary statistics and parameters is modeled, while BOLFI focuses on the relation between the parameters and the distance. A potential advantage of the latter approach is that the distance is a univariate quantity while the parameters and summary statistics may be multidimensional. Furthermore, BOLFI does not assume that the distance is defined via summary statistics and can be used without first running another ABC algorithm.

Learning of the model of $d(Y_\theta,{{y}}_0)$ requires data about the relation between $\theta$ and $d(Y_\theta,{{y}}_0)$. In BOLFI, the data are actively acquired focusing on regions of the parameter space where the distance tends to be small. This is achieved by leveraging techniques from Bayesian optimization (see e.g., Jones 2001; Brochu et al. 2010), hence its name. Ultimately, the framework provided by Gutmann and Corander (2016) reduces the computational cost of ABC by addressing both of the factors mentioned above. The first point is addressed by learning from data which parameter values tend to have small distances, whereas the second problem is resolved by focusing on areas where the distance tends to be small when learning the model and by not requiring further runs of the simulator once the model is learned.

While BOLFI is not restricted to a particular model for $d(Y_\theta,{{y}}_0)$, Gutmann and Corander (2016) used Gaussian processes in the applications in their paper. Gaussian processes have also been used in other work as surrogate models for quantities that are expensive to compute. Wilkinson (2014) used them to model the logarithm of $L(\theta)abc$, and the training data were constructed based on quasi-random numbers covering the parameter space. Meeds and Welling (2014) used Gaussian processes to model the empirical mean and covariances of the summary statistics as a function of $\theta$. Instead of simulating these quantities for every $\theta$, values from the model were used in a MCMC algorithm in approximating the likelihood. These approaches have been demonstrated to assist in speeding up ABC.

### Statistical Efficiency

We have seen that the statistical efficiency of ABC algorithms depends heavily on the summary statistics chosen, the distance between them, and the locality of the inference. In a recent work, (Gutmann et al. 2014) formulated the problem of measuring the distance between simulated and observed data as a classification problem: Two data sets are judged maximally similar if they cannot be told apart significantly above chance level (50% accuracy in the classification problem). On the other hand, two data sets are maximally dissimilar if they can be told apart with 100% classification accuracy. In essence, classification is used to assess the distance between simulated and observed data.

The classification rule used to measure the distance was learned from the data, which simplifies the inference since only a function (hypothesis) space needs to be prespecified by the user. In the process, Gutmann et al. (2014) also chose a subset or weighted (nonlinear) combination of summary statistics to achieve the best classification accuracy. This choice depended on the parameter values used to generate the simulated data. While computationally more expensive than the traditional approach, the classifier approach has the advantage of being a data-driven way to measure the distance between the simulated and observed data that respects the locality of the inference.

## Validation of abc

Due to the several levels of approximation, it is generally a recommendable practice to perform validatory analyses of the ABC inferences. We here discuss some of the possibilities suggested in the literature.

The ability to generate data from simulator-based models enables basic sanity checks for the feasibility of the inference with a given setting and algorithm. The general approach is to perform inference where synthetic data sets ${{y}}_0^*$ are generated with known parameter values $\theta^*$ to play the role of the observed data ${{y}}_0$. To assess whether the posterior distribution is concentrated around the right parameter values, one may then compute the average error between the posterior mean (mode) and $\theta^*$, or the expected squared distance between the posterior samples and $\theta^*$ (Wegmann et al. 2009). To assess whether the spread of the posterior distribution is not overly large or small, one may compute confidence (credibility) intervals and check their coverage. When the nominal confidence levels are accurate, 95% confidence intervals, for example, should contain $\theta^*$ in 95% of the simulation experiments (Wegmann et al. 2009; Prangle et al. 2014). Such tests can be performed *a priori* by sampling ${{y}}_0^*$ from the prior before having seen the actual data to be analyzed, or also *a posteriori* by sampling ${{y}}_0^*$ from the inferred posterior or from the prior restricted to some area of interest (Prangle et al. 2014). Corresponding techniques have also been suggested for the purpose of specifying the threshold value $\epsilon$ as discussed earlier in this article. It can be also beneficial here to store the generated data sets together with their parameter values so that the validations can be run without having to regenerate new data on every occasion.

The ABC framework provides a straightforward way to investigate the goodness-of-fit of the model. The distances $d(y_\theta,{{y}}_0)$ indicate how close the simulated data $y_\theta$ are to the observed data ${{y}}_0$. If all of the distances remain large, it may be an indication of a deficient model, as the model is unable to produce data similar to the observed data. Ratmann et al. (2009) proposed a method called ABC under model uncertainty (ABC$\mu$) where they augment the likelihood with unknown error terms for each of the different summary statistics used. The error terms are assumed to have mean zero and are sampled together with the parameters of the model. If, however, the mean of the error terms is found to deviate from 0, it may indicate a systematic error in the model.

Yet another issue is to consider identifiability of the model given the observed data. The likelihood function indicates the extent to which parameter values are congruent with the observed data. A strong curvature at its maximum indicates that the maximizing parameter value is clearly to be preferred, whereas a minor curvature means that several other parameter values are nearly equally supported by the data. More generally, if the likelihood surface is mostly flat over the parameter space, the data are not providing sufficient information to identify the model parameters. While the likelihood function is generally not available for simulator-based models, the arguments provided do also hold for the approximate likelihood function $L(\theta)abc$ in Equation (6). On one hand, the approximate likelihood function can be used to investigate the identifiability of the simulator-based model. On the other hand, it allows one to assess the quality of the distance $d$ or threshold $\epsilon$ chosen. Flat approximate likelihood surfaces, for instance, indicate that $\epsilon$ could be too large or that the distance function $d$ is not able to accurately measure differences between the data sets.

The approximate likelihood $L(\theta)abc$ can be obtained either by the method of Gutmann and Corander (2016) or also by any other ABC algorithm by assuming a uniform prior on a region of interest. Lintusaari et al. (2016) used such an approach to investigate the identifiability of the tuberculosis model considered as an example in the previous sections, and to compare different distance functions. Further, one may (visually) compare the (marginal) prior and the inferred (marginal) posterior (e.g., Blum 2010). Both approaches are applicable not only to the real observed data ${{y}}_0$ but also to the synthetic data ${{y}}_0^*$ for which the data-generating parameters $\theta^*$ are known. If the employed ABC algorithm is working appropriately, both $L(\theta)abc$ and the posteriors should clearly change when the characteristics of the observed data change markedly. In particular, if the number of observations is increased, the approximate likelihood and posterior should in general become more concentrated around the data-generating parameter values. While failure to pass such sanity checks may be an indicator that the choice of $d$ and $\epsilon$ could be improved, it can also indicate that the model may not be fully identifiable.

## Conclusion

It is possible to model complex biological phenomena in a realistic manner with the aid of simulator-based models. However, the likelihood function for such models is usually intractable and raises serious methodological challenges to perform statistical inference. ABC has become synonymous for approximate Bayesian inference for simulator-based models. We have here reviewed its foundations, the most widely considered inference algorithms, together with recent advances that increase its statistical and computational efficiency.

While the review is solely restricted to Bayesian methods, there exists a large body of literature on non-Bayesian approaches, for instance, the methods of simulated moments (McFadden 1989; Pakes and Pollard 1989) or indirect inference (Gouriéroux et al. 1993; Heggland and Frigessi 2004), both having their origin in econometrics.

We focused on the central topics related to parameter inference with ABC. Nevertheless, ABC is also applicable to model selection (see e.g., the review by Marin et al. 2012) and while we have reviewed methods making the basic ABC algorithms more efficient, we have not discussed the important topic of how to use ABC for high-dimensional inference. We point the interested readers to the work by Li et al. (2015) and also to the discussion by Gutmann and Corander (2016).

For practical purpose, there exist multiple software packages implementing the different ABC algorithms, summary statistic selection, validation methods, post processing, and ABC model selection methods. Nunes and Prangle (2015) provide a recent list of available packages with information about their implementation language, platform, and targeted field of study. In summary, ABC is currently a very active methodological research field and this activity will likely result in several advances to improve its applicability to answering important biological research questions in the near future.

## Appendix

For completeness, we state below the algorithms for MCMC ABC and SMC ABC by Marjoram et al. (2003) and Beaumont et al. (2009), respectively.
